# The Homoserine Dehydrogenase *thrA* Significantly Impairs Biofilm Formation, Stress Adaptation, and Virulence in *Klebsiella pneumoniae*

**DOI:** 10.3390/microorganisms14081751

**Published:** 2026-08-09

**Authors:** Wenqing Luo, Wangxin Wu, Yuanyi Zuo, Jing Zhu, Feng Zhang, Chuang Meng, Xinyu Miao, Tao Qin, Bangyue Zhou, Qingqing Gao, Daxin Peng, Yinyan Yin

**Affiliations:** 1 School of Basic Medical Sciences & School of Public Health, Faculty of Medicine, Yangzhou University, Yangzhou 225009, China; mx120231219@stu.yzu.edu.cn (W.L.); mx120251187@stu.yzu.edu.cn (W.W.); mx120231147@stu.yzu.edu.cn (J.Z.); mx120241163@stu.yzu.edu.cn (F.Z.); 2 Key Laboratory of the Jiangsu Higher Education Institutions for Nucleic Acid & Cell Fate Regulation, Yangzhou University, Yangzhou 225009, China; 3 College of Veterinary Medicine, Yangzhou University, Yangzhou 225009, China; mz120251718@stu.yzu.edu.cn (Y.Z.); miaoxy@yzu.edu.cn (X.M.); qintao@yzu.edu.cn (T.Q.); qqgao@yzu.edu.cn (Q.G.); 4 Jiangsu Key Laboratory of Zoonosis, Yangzhou University, Yangzhou 225009, China; mengchuang@yzu.edu.cn; 5 Jiangsu Interdisciplinary Center for Zoonoses and Biosafety, Yangzhou University, Yangzhou 225009, China; 6 Jiangsu Co-Innovation Center for Prevention and Control of Important Animal Infectious Diseases and Zoonoses, Yangzhou University, Yangzhou 225009, China; 7 Northern Jiangsu People’s Hospital, Clinical Medical College, Yangzhou University, Yangzhou 225001, China; abangnnn@163.com

**Keywords:** *Klebsiella pneumoniae*, biofilm, *thrA*, homoserine dehydrogenase, amino acid metabolism, virulence

## Abstract

*Klebsiella pneumoniae* (*K. pneumoniae*) is a major opportunistic pathogen associated with a broad spectrum of hospital-and community-acquired infections. Biofilm formation contributes to bacterial persistence, stress tolerance, and host immune clearance. In this study, we constructed a transposon mutant library of the clinical *K. pneumoniae* strain *KP*20 using a mariner-based mutagenesis system and screened for mutants exhibiting defective biofilm formation. Targeted knockout of *thrA* significantly reduced biofilm formation and attenuated the adhesion and invasion of *K. pneumoniae* with respect to human airway epithelial Calu-3 cells and lung adenocarcinoma A549 cells. These phenotypic changes are associated with altered transcription of the gene clusters responsible for type I and type III fimbria biosynthesis. Moreover, deletion of *thrA* compromised bacterial tolerance to acidic and alkaline stresses and increased susceptibility to phagocytosis by dendritic cells. In a murine infection model, *thrA* deletion significantly attenuated the virulence of *K. pneumoniae* and decreased bacterial colonization in target organs. Collectively, these findings indicate that *thrA* substantially contributes to biofilm formation, pH stress tolerance, host–cell interaction, and virulence in *K. pneumoniae*.

## 1. Introduction

*Klebsiella pneumoniae* (*K. pneumoniae*) is a prominent Gram-negative bacterium in the family Enterobacteriaceae and a leading causative agent of healthcare-associated infections worldwide. As an opportunistic pathogen, *K. pneumoniae* frequently colonizes human mucosal surfaces, including the oral mucosa and respiratory and gastrointestinal tracts [1]. When host immunity is compromised, these commensal populations can shift to a pathogenic state, triggering severe clinical manifestations such as pneumonia, urinary tract infections, bacteremia, and pyogenic liver abscesses. Previous studies have confirmed that *K. pneumoniae* can form biofilms on the surfaces of medical devices [2]. During device implantation, the pathogen can disseminate systemically via contaminated carriers, making it one of the most difficult pathogens to eliminate in clinical practice.

Biofilms are highly structured multicellular communities formed by bacteria as an adaptive strategy against hostile environments. They consist of an extracellular matrix (ECM) comprising polysaccharides, proteins, nucleic acids, and host-derived components. This structure provides a protective microenvironment that shields bacteria from host immune clearance and confers high-level tolerance to conventional antibiotics [3]. *K. pneumoniae* readily forms biofilms on implantable medical devices, which not only increases the risk of severe complications in patients but also prolongs the course of disease and increases the overall treatment cost burden [4]. Therefore, it is critical to identify the genetic determinants governing the transition from a planktonic to a biofilm-associated lifestyle in *K. pneumoniae*.

Transposon mutagenesis is a powerful and widely used functional genomics approach to identifying bacterial genes associated with specific phenotypic traits. The core principle relies on the random integration of transposons into the bacterial genome, resulting in disruption of target gene function and generation of diverse mutant libraries [5]. After phenotypic screening for the desired traits, genomic sequencing combined with bioinformatic analysis enables the precise identification of genetic loci responsible for the phenotypic changes observed [6]. This technique is commonly applied to characterize physiological processes such as biofilm formation, host–cell adhesion, and serum resistance. Owing to its high efficiency, transposon mutagenesis has been extensively employed in diverse bacterial species for the discovery and functional characterization of specific genes [7].

Deciphering the molecular regulatory networks governing biofilm formation is critical to address the clinical threats posed by *K. pneumoniae*. Although type I (*fim*) and type III (*mrk*) pili have been well documented as pivotal determinants modulating the biofilm formation capacity of *K. pneumoniae* [8], other novel genetic regulators involved in this process remain largely uncharacterized. In this study, approximately 600 insertion mutants of the *KP*20 strain were constructed via mariner-based random transposon mutagenesis. Subsequent quantitative screening using a 96-well crystal-violet staining assay yielded four biofilm-deficient mutant strains, among which the *thrA* gene was selected for further mechanistic investigation. The *thrA* gene encodes homoserine dehydrogenase (HSD), a component of the bifunctional aspartokinase/homoserine dehydrogenase complex and a key enzyme in the aspartate metabolic pathway. It is well-established that under amino-acid-limited conditions, this enzyme plays a crucial role in the growth and metabolic processes of *Mycobacterium tuberculosis* [9]. Previous studies have also proven that *thrA* deletion markedly impairs the in vitro growth of *M. tuberculosis* in amino-acid-limited media and abolishes its pathogenicity in murine infection models. Nevertheless, the potential involvement of this core metabolic gene in biofilm maturation and virulence regulation in *K. pneumoniae* still remains poorly described.

Following the construction of a *thrA* mutant and complemented strains, we performed an extensive phenotypic evaluation. The assays included growth curve analysis performed to rule out intrinsic metabolic defects, capsular staining employed to evaluate ECM production, and antimicrobial susceptibility testing. Furthermore, host–pathogen interaction phenotypes were assessed via epithelial cell adhesion and invasion assays as well as dendritic cell phagocytosis experiments. In addition, a murine infection model was utilized to explore the regulatory role of *thrA* in bacterial virulence and bacterial loads in representative target organs, including the lung and spleen. Collectively, this study identifies *thrA* as a crucial regulator of biofilm formation and in vivo pathogenicity in *K. pneumoniae*, providing novel mechanistic insights into the molecular basis of biofilm-associated infections caused by this pathogen.

## 2. Materials and Methods

### 2.1. Animal Ethics Statement

All animal experiments were performed in accordance with the Jiangsu Administrative Committee for Laboratory Animals and have been approved by it (Permission number: 202309831).

### 2.2. Bacterial Strains, Plasmids, and Culture Conditions

All strains and plasmids utilized in this study are listed in Table 1. The well-characterized virulent *K. pneumoniae* strain *KP*20 was used as the wild-type (WT) strain [10]. This strain was selected for subsequent functional characterization on account of its robust biofilm-forming capacity, marked virulence, and frequent recovery from clinical specimens [10].

Plasmid pBT20 was used for the construction of the transposon mutant library. *Escherichia coli* (*E. coli*) strains DH5α and S17-1 λpir served as the hosts for molecular cloning and bacterial conjugation experiments, respectively. All bacterial strains were cultured in Luria–Bertani (LB) broth at 37 °C with shaking at 220 r/min. For selective cultivation, antibiotics were supplemented at the following concentrations: chloramphenicol (30 μg/mL) and ampicillin (60 μg/mL) [11].

**Table 1:** Bacterial strains and plasmids used in this study.

Strain or Plasmid	Characteristics	Source or References
*KP*20	*K. pneumoniae* ST23 strain isolated from a patient’s sputum	This study
*KP*20 Δ*thrA*	*KP*20 mutant with the *thrA* gene deleted	This study
*KP*20 Δ*thrA*-compl	Complementation of *KP*20 Δ*thrA*	This study
DH5α	*endA1 hsdR*17 (rk^−^mk^+^)supE44 thi-1 *recA1 gyrA* (NalR) RelA1Δ (*lacIZYA*-*argF*) U169deoR (ф80d lac Δ(lacZ) M15)	Shanghai Weidi Biotechnology Co., Ltd. (Shanghai, China)
S17-1 λpir	F^−^, *thi*, *pro*, *hsdR*, *hsdM*^+^, *recA*[chr::*RP4*-*2-*Tc::Km::*Tn7*]	Shanghai Weidi Biotechnology Co., Ltd.
pBT20	Mariner transposon mutagenesis vector, Amp^R^ Gm^R^	[12]
pKD46	Expresses λ Red recombinase, Amp^R^	[12]
pKD46-Kan	A derivative of pKD46 wherein the ampicillin resistance gene is replaced by a kanamycin resistance gene	[12]
*KP*20-pKD46-Kan	The *KP*20 carrying recombinant pKD46 with the ampicillin resistance gene replaced by a kanamycin resistance gene, for convenient mutant screening.	[12]
pDM4	Suicide plasmid, *sacB*, mobRP4, oriR6K, Cm^R^	[12]
pACYC184	Complementary vector, Cm^R^ Tc^R^	[12]

### 2.3. Transposon m2.3. Transposon Mutagenesis and Screening for Biofilm-Deficient Mutants

A mariner-based transposon mutant library was generated in *KP*20 using the pBT20 transposon delivery system, as previously described [13]. Briefly, *Escherichia coli* S17-1 λpir carrying pBT20 was used as the donor strain, and *KP*20-pKD46-Kan was used as the recipient strain. Transconjugants were selected on antibiotic-containing LB agar plates and individually screened for reduced biofilm formation using a 96-well crystal-violet (CV, Beijing Solarbio Science & Technology Co., Ltd., Beijing, China) assay under static culture conditions [14]. Mutants showing significantly lower OD_550_ values than *KP*20 were selected for insertion-site identification. Detailed mating, selection, and screening procedures are provided in Supplementary Methods S1.

### 2.4. Identification of Transposon Insertion Sites

Following Chen et al.’s method [15], we identified transposon insertion sites in biofilm-deficient mutants via arbitrarily primed PCR followed by Sanger sequencing, as previously described. Two rounds of PCR were performed using the transposon-specific and arbitrary primers listed in Table 2. The purified second-round PCR products were sequenced using primer P7-2, and the insertion sites were mapped to the *KP*20 genome. The details of the PCR conditions are provided in Supplementary Methods S2.

### 2.5. Construction of thrA Deletion and Complemented Strains

An in-frame deletion of *thrA* was generated in *KP*20 via suicide-plasmid-mediated homologous recombination, as previously described [16]. Candidate deletion mutants were screened via antibiotic selection and confirmed via PCR and Sanger sequencing [17]. For complementation, the full-length *thrA* coding sequence, together with its putative native promoter region, was cloned into pACYC184 and introduced into *KP*20 Δ*thrA*, generating *KP*20 Δ*thrA*-compl. Chloramphenicol was used only during bacterial culture before inoculum preparation to maintain the recombinant plasmid in the complemented strain. It was not added to the mammalian cell culture medium or administered to the mice during the infection experiments. The details of the cloning, screening, and verification procedures are provided in Supplementary Methods S3, and the primers used are listed in Table 2. SYBR Green-based real-time quantitative reverse transcription PCR (qRT-PCR) was performed to quantify *thrA* transcription level and validate the complemented strain (Supplementary Methods S4). The primers used are listed in Table 2.

### 2.6. Growth Kinetics and In Vitro Stress Tolerance Assay

The overnight cultures of *KP*20, *KP*20 Δ*thrA*, and *KP*20 Δ*thrA-*compl were diluted to an initial OD_600_ of 0.1 in 10 mL of LB liquid medium and incubated at 37 °C with shaking at 220 r/min. Bacterial OD_600_ values were continuously monitored for 8 h, and bacterial growth curves were plotted according to the hourly growth data for each strain.

The bacterial culture in the logarithmic phase was washed with sterile phosphate-buffered saline (PBS), and then serial dilutions were performed to determine the original colony count [18]. The bacterial suspensions were subsequently exposed to acid, alkaline, hypertonic, and oxidative stress conditions for 30 min. After stress treatment, viable bacteria were re-enumerated, and the bacterial survival rate was calculated as the ratio of post-stress viable counts to the initial viable bacterial counts.

We performed threonine restriction assays to observe phenotypic alterations in the *KP*20 Δ*thrA* under threonine-deprived conditions [19]. Three strains were cultured overnight in M9 medium. Auxotrophs were supplemented with 100 μM threonine to reach the stationary phase and a uniform starting biomass. Cultures were pelleted and washed twice with fresh M9 to eliminate residual amino acids. Cells were resuspended and diluted from OD_600_ = 0.1 for growth assays. A 10 mM threonine stock was prepared per manufacturer guidelines, with serial dilutions yielding final amino acid concentrations of 0, 15, 30, 50, 100, and 200 μM. The cultures were incubated at 37 °C with continuous shaking at 220 rpm for 12 h, after which the OD_600_ of each culture was measured.

### 2.7. Biofilm Assay

The biofilm formation ability of *KP*20, *KP*20 Δ*thrA*, and *KP*20 Δ*thrA*-compl were tested using the 96-well method. The strains were incubated for 12 h at 37 °C with shaking at 220 r/min. The overnight cultures were adjusted to an initial OD_600_ of 0.8. For the 96-well assays, each well contained 180 μL of 1:10-diluted Nutrient Broth plus 20 μL of standardized bacterial suspension. Plates were incubated at 37 °C for 24 h; then, OD_600_ measurement and washing with ddH_2_O were performed. Subsequently, 200 μL of 0.4% CV solution was added to each well and incubated at room temperature for 30 min. Then, the plates were washed three times with double distilled water (ddH_2_O) and stained for 30 min. For quantitative analysis, the CV retained in biofilm matrices was completely solubilized using 200 μL of absolute ethanol per well, and the absorbance at 550 nm was subsequently measured. The obtained OD_550_ values reflected the relative biofilm formation levels, enabling quantitative comparison of biofilm production among the three strains. All experiments were performed in triplicate to ensure reproducibility instead.

*K. pneumoniae* strains were inoculated into LB broth and incubated for 12 h at 37 °C and 220 r/min. After centrifugation at 4 °C and 5000× *g*, bacterial cells were resuspended in 500 μL sterile LB medium and mixed 1:1 with fresh LB medium in a sterile 24-well plate. The culture system was incubated statically at 37 °C for 24 h. Then, biofilms were fixed with 2.5% glutaraldehyde for 12 h. After a wash with PBS, the bacterial samples were subjected to gradient dehydration using a series of anhydrous ethanol concentrations (30%, 50%, 70%, 90%, 95%, and 100%), with each dehydration treatment lasting 10–15 min. The processed samples were subjected to critical-point drying and gold sputtering. The processed slides were mounted on a specimen stage and observed using a Zeiss Gemini SEM 300 field emission scanning electron microscope (Carl Zeiss, Oberkochen, Germany) to examine the morphology of the bacterial biofilms [20].

### 2.8. String Test and Mucoviscosity Assay

The strains were plated on blood agar and incubated overnight at 37 °C for 48 h [21]. A single colony was gently dabbed and streaked outward using an inoculating loop. A mucoviscous string longer than 5 mm was considered a positive result, indicating that an isolate has high mucoviscosity. The mucoviscosity assay for *KP*20, *KP*20 Δ*thrA*, and *KP*20 Δ*thrA-*compl was performed using the method described above [22].

### 2.9. Detection of Capsule and Fimbriae

Capsule staining was performed as described previously [23]. Briefly, a 5% (*w*/*v*) Congo red solution was prepared by dissolving 5 g of Congo red powder in 100 mL of distilled water. The Congo red (Sangon Biotech, Shanghai, China) solution was mixed with serum (Gibco, New York, NY, USA) in a 5:1 ratio, and the mixture was placed at one end of a clean glass slide. Bacterial colonies were picked from agar plates using an inoculating loop and thoroughly mixed with the staining mixture. Referring to the blood smear preparation protocol, we spread the staining mixture such that it was deposited as a thin film on the slide and air-dried it at room temperature. The dried film was then covered with 5% dilute hydrochloric acid for 1 min and rinsed under running tap water to remove excess acid. Subsequently, crystal-violet staining solution was applied to the film for 1 min, and the slide was rinsed again with running water and air-dried. Finally, the capsule thickness of the bacteria was observed and recorded under a light microscope.

To analyze the morphological structure of the bacterial capsule, bacterial samples were fixed using the lysine–acetylformaldehyde/glutaraldehyde–ruthenium red–osmium (LRR) method. Briefly, bacterial cells were harvested via centrifugation and fixed in ice-cold sodium dimethylarsenate buffer supplemented with 0.075% ruthenium red (Yuanye Biotech, Shanghai, China) and 0.075 M lysine acetate for 20 min. After being washed with potassium hydroxide buffer containing 0.075% ruthenium red, the samples were subjected to secondary fixation for 24 h, followed by another round of washing with the same potassium hydroxide buffer). Subsequently, the processed samples were sent to Wuhan BioSys Co., Ltd. (Wuhan, China), for ultrathin-section preparation. The capsular structures of the samples were then observed and imaged under a transmission electron microscope (TEM).

Two milliliters of overnight culture was centrifuged at 4 °C and 1000× *g* for 10 min. After three PBS washes, the pellets were sent to the Yangzhou University Testing Center. Negative staining was performed prior to TEM observation of the bacterial surface features.

### 2.10. Antimicrobial Susceptibility Test

Antimicrobial susceptibility was determined using the Kirby–Bauer disk diffusion method, with the results interpreted according to the Clinical and Laboratory Standards Institute guidelines. A total of 19 antibiotics were tested: chloramphenicol, amikacin, ciprofloxacin, doxycycline, gentamicin, ampicillin, kanamycin, norfloxacin, cefoxitin, meropenem, imipenem, aztreonam, ofloxacin, tetracycline, minocycline hydrochloride, streptomycin, ceftriaxone sodium, cefixime, and cefazolin. *E. coli* ATCC25922 was used as a quality-control strain.

### 2.11. Adhesion and Invasion Assays Regarding K. pneumoniae and Calu-3 and A549 Cells

In vitro adhesion and invasion assays regarding *K. pneumoniae* were performed as described previously [24]. Briefly, Calu-3 and A549 cells were seeded into 24-well plates and cultured until reaching 80% confluence prior to infection. The cells were infected with bacteria at a multiplicity of infection (MOI) of 100 and incubated at 37 °C in a 5% CO_2_ incubator for 1 h [12,25]. After incubation, we removed unattached bacteria by washing the cells three times with sterile PBS. The cells were treated with 100 μL of 1% Triton X-100. The number of adherent bacteria was quantified via serial dilution of the cell lysate and plating the result onto LB agar plates, followed by colony counting after incubation. For the invasion assay, after 1 h of infection, the cells were further cultured in Dulbecco’s Modified Eagle Medium (DMEM, Gibco, New York, NY, USA) supplemented with 100 μg/mL of gentamicin for 1 h to eliminate extracellular bacteria. Subsequently, the cells were washed three times with PBS and lysed with 1% Triton X-100, and the cell lysate was serially diluted and plated onto LB agar plates for colony counting.

For confocal laser-scanning microscopy (CLSM) observation, Calu-3 cells were cultured as a monolayer on sterile coverslips and infected with bacteria at an MOI of 100 for 1 h. After infection, the cells were washed three times with PBS, fixed with 4% paraformaldehyde, and treated with 0.4% Triton X-100 for 10 min. Non-specific binding sites were blocked with 0.5% bovine serum albumin (BSA, Bioolook, Nanjing, China) for 30 min at room temperature. The cells were then incubated with a *KP*20 polyclonal antibody (1:250 dilution, produced by immunizing mice) overnight at 4 °C. After being washed with PBS, the cells were incubated with Alexa Fluor 488-conjugated goat anti-mouse IgG (1:200, Abcam, Cambridge, England) for 1 h at room temperature in the dark. Nuclei were stained with 4′,6-diamidino-2-phenylindole (DAPI, Abcam, Cambridge, UK) for 10 min. The samples were observed using a Nikon confocal laser-scanning microscope (Tokyo, Japan).

### 2.12. Assay for the Phagocytosis of K. pneumoniae by Dendritic Cells (DCs)

To evaluate the impact of the *thrA* gene on bacterial phagocytosis by DCs, we isolated bone marrow DCs from specific-pathogen-free (SPF) female C57BL/6 mice (6–8 weeks old) [26]. DCs were cultured in RPMI 1640 medium supplemented with 10% fetal bovine serum (FBS, Gibco, New York, NY, USA), 1% granulocyte-macrophage colony-stimulating factor (GM-CSF, Peprotech, Rocky Hill, CT, USA), and 1% interleukin-4 (IL-4, Peprotech, Rocky Hill, CT, USA) at 37 °C in a 5% CO_2_ incubator. Bacterial strains were added at an MOI of 100 [27], and the co-cultures were incubated at 37 °C for 0.5 h and 2 h, respectively. After infection, the cells were washed three times with sterile PBS and then incubated with fresh medium containing 100 μg/mL of gentamicin for 1 h to eliminate extracellular bacteria. Then, the DCs were lysed with 0.5% Triton X-100 for 10 min at room temperature. The cell lysate was plated onto the LB agar plates and incubated at 37 °C overnight.

### 2.13. Pathogenicity of K. pneumoniae in Mouse Infection Model

To evaluate the virulence of *K. pneumoniae* strains *KP*20, *KP*20 Δ*thrA*, and *KP*20 Δ*thrA*-compl, a murine pneumonia infection model was established as described previously [10]. Specific pathogen-free (SPF) C57BL/6 female mice (6–8 weeks old) were used in this study. Five mice were randomly assigned to each cage, with food and water provided. After being weighed, the mice were anesthetized with an intraperitoneal injection of tribromoethanol. Prior to infecting the mice, each prepared bacterial suspension was serially diluted and spread onto LB agar in triplicate for each sample. After incubation, the colony-forming units were counted to accurately verify the actual dose of live bacteria injected into each mouse in each dilution group. Then, each mouse was intratracheally injected with either 20 μL of bacterial suspension or sterile PBS. After injection, the incision site was sealed with tissue adhesive. Bodyweight was measured daily for the subsequent 15 days, and the survival rate was recorded. The median lethal dose (LD_50_) and 95% confidence interval (95% CI) were calculated using a modified version of Karber’s method. After the experiment, all remaining mice were euthanized with CO_2_, sealed in dedicated packaging, cryopreserved, and transported to the Animal Carcasses Harmless Treatment Center of Yangzhou University for pollution-free treatment.

Then, 24 h post-infection (inoculation dose: 1 × 10^4^ CFU), five mice per group were randomly euthanized. Spleen and lung tissues were harvested, weighed, and homogenized in sterile PBS, and the homogenates were inoculated onto LB agar plates for bacterial colony counting. Additionally, bronchoalveolar lavage fluid (BALF) was collected at 24 h post-infection, and the levels of tumor necrosis factor (TNF)-α, interleukin (IL)-6, IL-10, and transforming growth factor (TGF)-β in the BALF were determined using an enzyme-linked immunosorbent assay (ELISA) kit (Lianke bio, Hangzhou, Zhejiang, China) in accordance with the manufacturer’s instructions. At 24 h post-infection, lung and spleen tissues were also collected, fixed in 4% formaldehyde, embedded in paraffin, sectioned, and stained using a hematoxylin–eosin (HE) assay for histopathological examination under a light microscope. Two pathologists independently evaluated the histopathological lesions.

At 24 h post-infection with a dose of 1 × 10^4^ CFU, lung and spleen tissues were isolated and immediately placed in 4% paraformaldehyde solution for fixation. After 24 h of fixation, the tissues were embedded and subjected to Optical Coherence Tomography (OCT, Torrance, CA, USA) to prepare frozen sections. The sections were treated with 0.4% Triton X-100 solution for 5 min at room temperature and then blocked with 5% BSA for 1 h. The sections were then incubated overnight at 4 °C with *KP*20 polyclonal antibody (1:250 dilution, produced by immunizing mice). After being washed with sterile PBS, the sections were incubated with Alexa Fluor 488-conjugated goat anti-mouse IgG (1:200 dilution) for 1 h at room temperature in the dark. Subsequently, DAPI was used to stain nuclei. The sections were observed using a Nikon confocal laser-scanning microscope (Nikon, Tokyo, Japan).

The histopathological lesions were scored independently by two experienced pathologists under blinded conditions; examiners were unaware of sample genotypes. Briefly, a 0–3 scoring scale was adopted (0 = normal, 1 = mild lesion, 2 = moderate lesion, 3 = severe lesion). All mice were euthanized using CO_2_ and collectively transported to the animal carcass disposal center at Yangzhou University for harmless treatment.

### 2.14. Quantification of Transcript Levels for Biofilm-Related Genes

RNA was extracted from bacteria by using the TRIzol method. The mRNA transcript levels of bacterial biofilm-associated genes were detected using qRT-PCR. The extracted total RNA was reverse-transcribed into complementary DNA (cDNA) using the TransScript All-in-One First-Strand cDNA Synthesis SuperMix for qPCR (TRANS, Beijing, China). Quantitative PCR analysis was performed with PerfectStart Green qPCR SuperMix (Bioer Technology, Hangzhou, China) in accordance with the manufacturer’s instructions. The thermal cycling parameters were set as follows: pre-denaturation at 94 °C for 30 s, followed by 45 amplification cycles consisting of denaturation at 94 °C for 5 s and annealing/extension at 60 °C for 30 s. The *16S* rRNA gene was used as the housekeeping gene for internal normalization. The relative mRNA expression levels were calculated using the 2^−ΔΔCt^ method and expressed as the relative fold change compared with the control group [28]. The sequences of the primers used in this study are listed in Table 2.

### 2.15. Statistical Analysis

All data were analyzed with GraphPad Prism 8 (San Diego, CA, USA) and expressed as means ± standard deviations (SDs). One-way ANOVA was applied for intergroup comparisons, with LSD, Bonferroni, or Tamhane’s T2 post hoc tests adopted for multiple comparisons based on homogeneity of variance. Asterisks denote statistical significance: * *p* < 0.05, ** *p* < 0.01.

## 3. Results

### 3.1. Identification of Biofilm-Deficient Mutants

**Figure 1:**
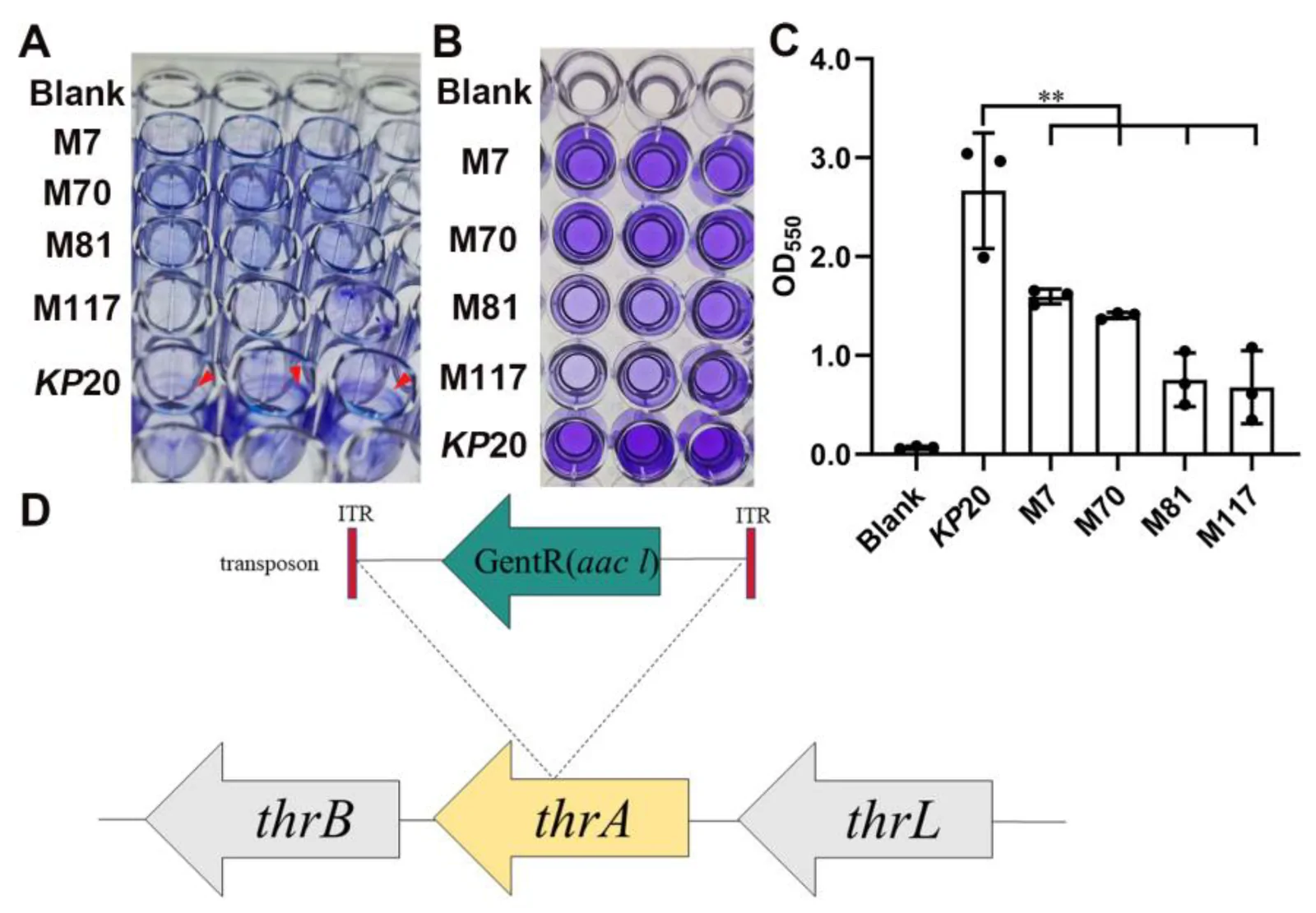
Identification of mutants with reduced biofilm formation capacity in *KP*20 via transposon mutagenesis. (**A**,**B**) Representative images of biofilm formation in *KP*20 transposon insertion mutants in 96-well plates. Overnight cultures were diluted in 1/10 Nutrient Broth, and 200 μL of each bacterial suspension was added to a 96-well-plate and then incubated at 37 °C for 24 h. Results were observed after staining with 0.4% crystal violet. (**A**) Images of biofilm formation on the wall of the 96-well plate (indicated by the red arrow). (**B**) Images of crystal violet dissolved with ethanol. (**C**) OD_550_ measurements of *KP*20 transposon insertion mutants after crystal-violet staining. (**D**) Genomic location of the transposon insertion in the *thrA* gene of the M70 mutant. Data represent means ± SDs of three independent experiments. Pairwise comparisons were conducted between *KP*20 and M7, M70, M81, and M117. ** *p* < 0.01.

Screening of approximately 600 transposon mutants yielded four strains with markedly impaired biofilm formation relative to *KP*20 (Figure 1A–C). Two rounds of PCR were performed for sample validation. No target amplicon was obtained from strain M7 in either round, precluding Sanger sequencing. In contrast, M70, M81, and M117 yielded amplicons in the second PCR round and were subjected to Sanger sequencing; corresponding electrophoresis results are shown in Figure S2. Sequencing verified transposon insertion within *thrA* in mutant M70 and independent insertions in *ornD* in M81 and M117 (Table 3, Figure 1D). Given its striking biofilm-deficient phenotype and greater novelty, *thrA* was chosen for subsequent targeted functional characterization.

### 3.2. Characterization of the thrA Deletion Mutant

**Figure 2:**
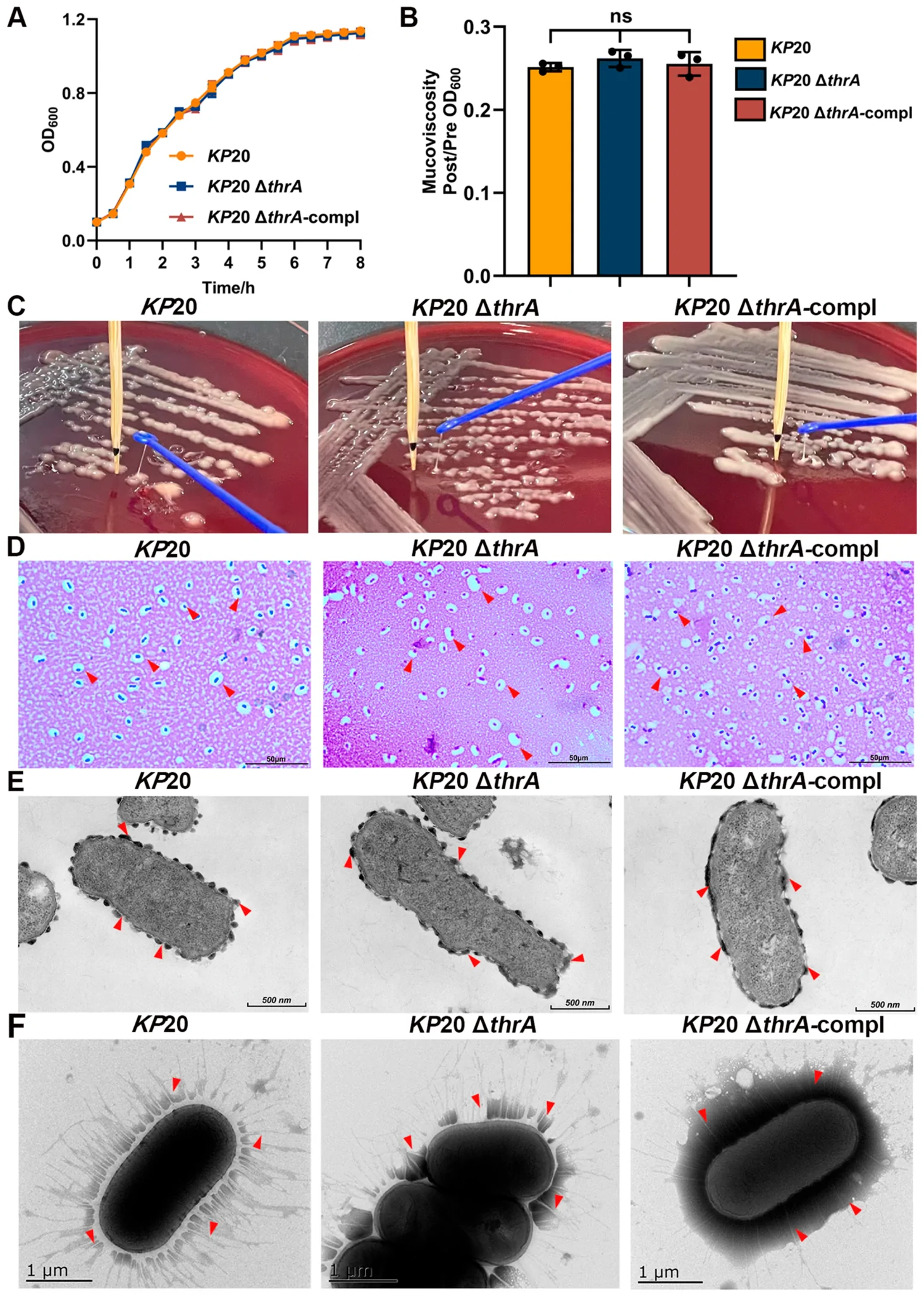
Growth kinetics, mucoviscosity, and capsule characterization of *KP*20, *KP*20 Δ*thrA*, and *KP*20 Δ*thrA*-compl. (**A**) Bacterial growth curves in LB broth at 37 °C and 220 r/min. (**B**) Quantitative mucoviscosity was determined via centrifugation assay. Pairwise comparisons were conducted between *KP*20 Δ*thrA* and *KP*20 and *KP*20 Δ*thrA* and *KP*20 Δ*thrA*-compl (ns: *p* > 0.05). (**C**) Representative images of the string test (a string length > 5 mm indicates a positive result). (**D**) Light microscopy images of capsule staining using Congo red and crystal violet; the capsule is indicated by the red arrow (bars: 50 µm). (**E**) TEM images showing the ultrastructural morphology and thickness of the bacterial capsule; the capsule is indicated by the red arrow (bars: 500 nm). (**F**) TEM image shows the ultrastructural morphology of bacterial fimbriae; the red arrows point to the fimbriae. Scale bar: 1 µm. Data represent means ± SDs of three independent experiments.

To investigate whether the *thrA* gene affects the growth capacity of *K. pneumoniae* strain *KP*20, we compared the growth curves of *KP*20, *KP*20 Δ*thrA*, and *KP*20 Δ*thrA*-compl in LB medium. The optical density at 600 nm (OD_600_) of the bacterial cultures in LB medium was measured hourly. As shown in Figure 2A, we observed no significant differences in the growth profiles among the three strains. Subsequently, we performed mucoviscosity measurement, a string test, and capsule staining and made TEM observations of capsule formation to assess whether *thrA* regulates capsular phenotypes. The results showed that there were no significant differences in mucoviscosity measurement between *KP*20 and its derivative strains (Figure 2B). In the string test, all three strains produced long, viscous strings, indicative of a consistent hyper-mucoviscous phenotype (Figure 2C). Consistent with these observations, both capsule staining and TEM analysis showed comparable capsular morphology and thickness across the three strains (Figure 2D,E). Representative TEM micrographs revealed no obvious qualitative differences in the overall morphology of surface appendages between *KP*20 Δ*thrA* and *KP*20 (Figure 2F). However, as fimbrial density was not quantified, moderate or fimbrial-type-specific alterations cannot be excluded. Collectively, no significant differences in growth, mucoviscosity, capsular morphology, or fimbrial structures were observed among the three strains.

### 3.3. Antimicrobial Susceptibility of the thrA Deletion Mutant

To determine whether the *thrA* gene contributes to antimicrobial resistance in *K. pneumoniae*, the antibiotic susceptibility profiles of *KP*20, *KP*20 Δ*thrA*, and *KP*20 Δ*thrA*-compl were assessed against 19 clinically relevant antibiotics via the Kirby–Bauer (K-B) disk diffusion method (Microbial Reagent, Hangzhou, China). As summarized in Table 4, both the *KP*20 strain and *KP*20 Δ*thrA* exhibited resistance to four antibiotics: tetracycline, doxycycline, minocycline, and ampicillin. The *KP*20 Δ*thrA*-compl displayed identical resistance to these four antibiotics as well as acquired chloramphenicol resistance, which was attributed to the chloramphenicol resistance cassette carried by the pACYC184 complementation vector. Apart from vector-associated chloramphenicol resistance, the three strains showed comparable antimicrobial susceptibility profiles.

### 3.4. Stress Tolerance and Amino Acid Restriction Assays Regarding the thrA Deletion Mutant

**Figure 3:**
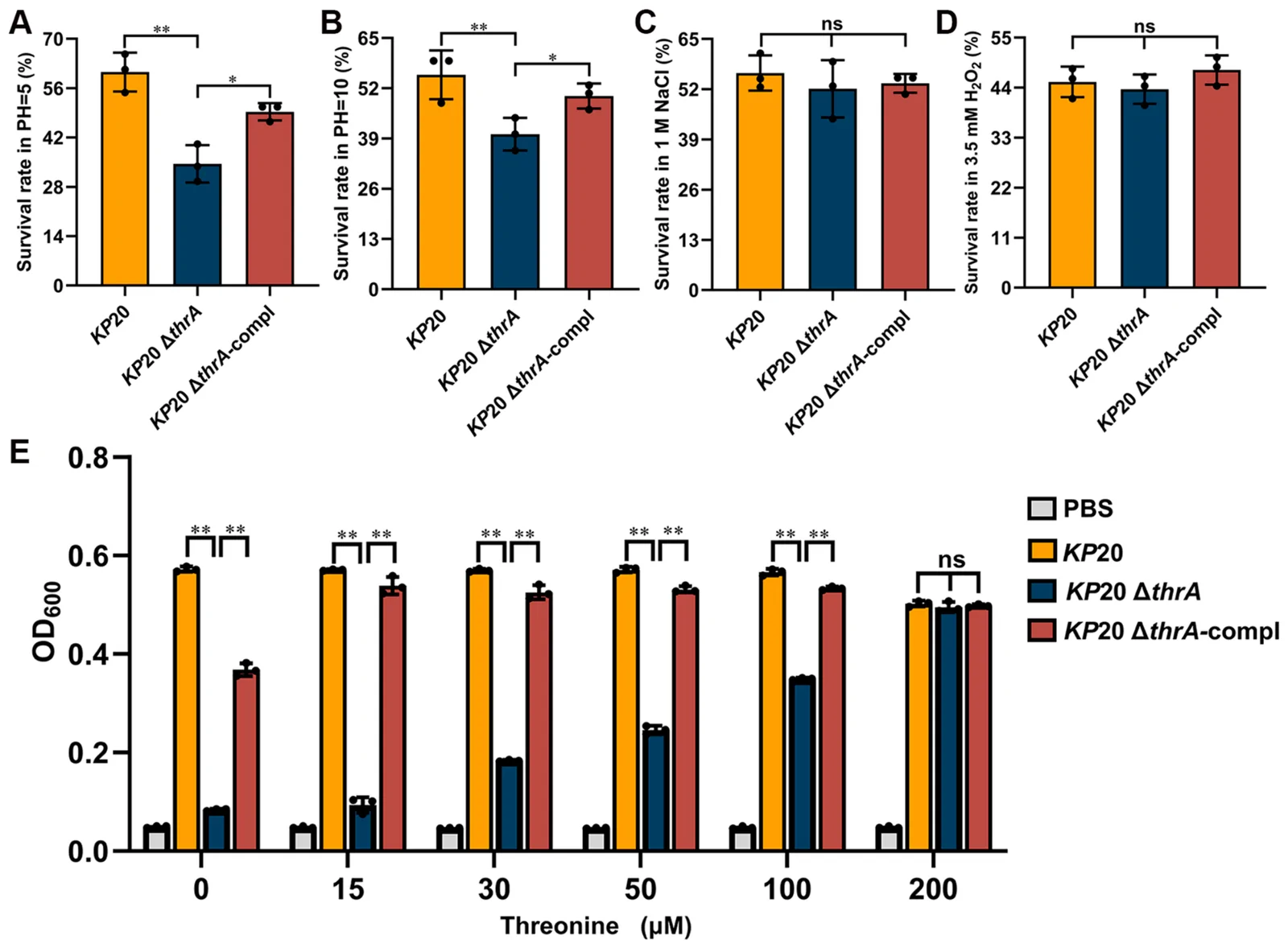
The survival rates of *KP*20, *KP*20 Δ*thrA*, and *KP*20 Δ*thrA*-compl under different stress conditions. (**A**) Acidic, (**B**) alkaline, (**C**) hyperosmotic, and (**D**) oxidative stress conditions. (**E**) Standardized M9 medium with serially increased threonine concentrations was loaded into a 96-well microplate. Continuous OD_600_ monitoring was performed during cultivation, and OD_600_ values were measured at 12 h. The data represent means ± SDs of three independent experiments. Pairwise comparisons were conducted between *KP*20 Δ*thrA* and *KP*20 and *KP*20 Δ*thrA* and *KP*20 Δ*thrA*-compl, ** *p* < 0.01, * *p* < 0.05, ns: *p* > 0.05.

The survival of *KP*20, *KP*20 Δ*thrA*, and *KP*20 Δ*thrA*-compl was compared after 30 min exposure to acidic, alkaline, hyperosmotic, and oxidative stress conditions. As shown in Figure 3A,B, *KP*20 Δ*thrA* exhibited significantly lower survival rates under both acidic and alkaline conditions compared to *KP*20 (*p* < 0.01). This phenotype was fully reversed in the complemented strain, which showed viability levels comparable to those of *KP*20. In contrast, no significant differences in survival rate were observed among the three strains when exposed to hyperosmotic or oxidative stress (Figure 3C,D). These results indicate that *thrA* contributes to the survival of *KP*20 under acidic and alkaline stress.

The results of amino acid limitation assays revealed that the *thrA* deletion mutant displayed pronounced growth repression under threonine deprivation (Figure 3E). Its growth recovered progressively with an increase in exogenous threonine, and 200 μM represented the minimal supplementation concentration sufficient to restore *KP*20 growth phenotypes.

### 3.5. Biofilm Phenotype of the thrA Deletion Mutant

**Figure 4:**
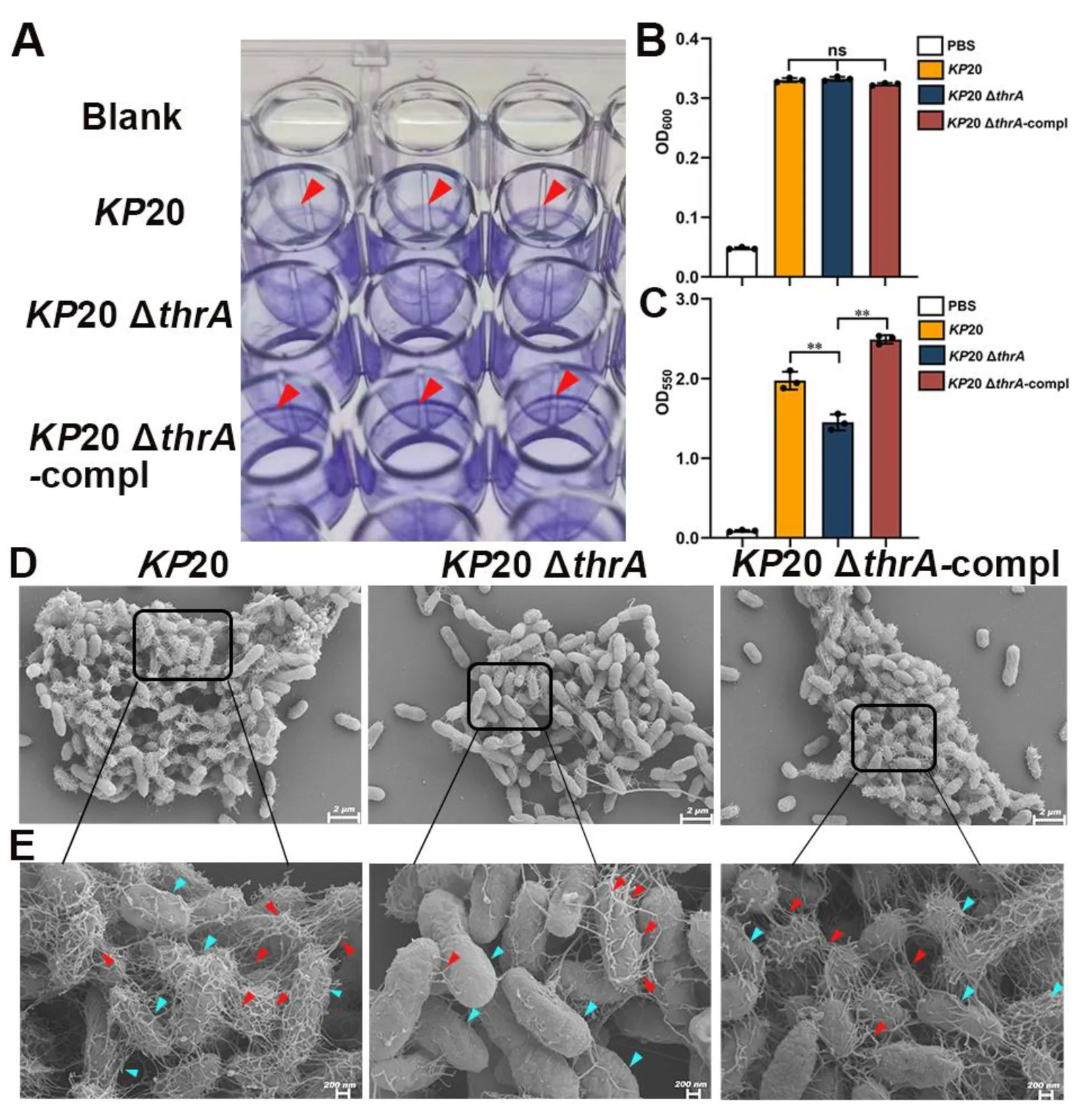
Biofilms of *KP*20, *KP*20 Δ*thrA*, and *KP*20 Δ*thrA*-compl. (**A**) Crystal-violet staining of bacterial biofilms grown on 96-well-plates (red arrow indicating). A total of 200 μL of each bacterial suspension was incubated at 37 °C for 24 h and stained with 0.4% crystal violet. (**B**) Following 24 h of static incubation of *K. pneumoniae* at 37 °C, absorbance was measured at 600 nm. (**C**) Quantitative analysis of biofilm formation based on absorbance at 550 nm (OD_550_). (**D**) The bacterial biofilm was observed using a scanning electron microscope (bars: 2 µm). (**E**) The biofilm matrix is indicated by the red arrow, and the bacteria are indicated by the blue arrow (bars: 200 nm). The data represent means ± SDs of three independent experiments. Pairwise comparisons were conducted between *KP*20 Δ*thrA* and *KP*20 and *KP*20 Δ*thrA* and *KP*20 Δ*thrA*-compl, ns: no significance, ** *p* < 0.01.

To further determine whether *thrA* deletion affects biofilm formation in *KP*20, we evaluated the three strains using a 96-well CV assay. CV staining revealed that while the *KP*20 and *KP*20 Δ*thrA*-compl strains formed robust, thick biofilms, *KP*20 Δ*thrA* exhibited significantly weaker biofilm formation (Figure 4A). Quantitative analysis confirmed this phenotype; no significant differences in bacterial growth (OD_600_) were found among the three groups (Figure 4B and Figure S1). The absorbance (OD_550_) of the *KP*20 Δ*thrA* biofilm (1.45 ± 0.06) was significantly lower than that of *KP*20 (1.97 ± 0.06) and *KP*20 Δ*thrA*-compl (2.49 ± 0.03) (Figure 4C). SEM further showed that *KP*20 Δ*thrA* formed a less dense biofilm matrix than *KP*20 and the complemented strain (Figure 4D,E). The SEM observations were consistent with the quantitative results obtained from the 96-well plate crystal-violet staining assay. Collectively, the *thrA* gene substantially contributes to robust biofilm in *K. pneumoniae*.

### 3.6. Host–Cell Interactions of the thrA Deletion Mutant

**Figure 5:**
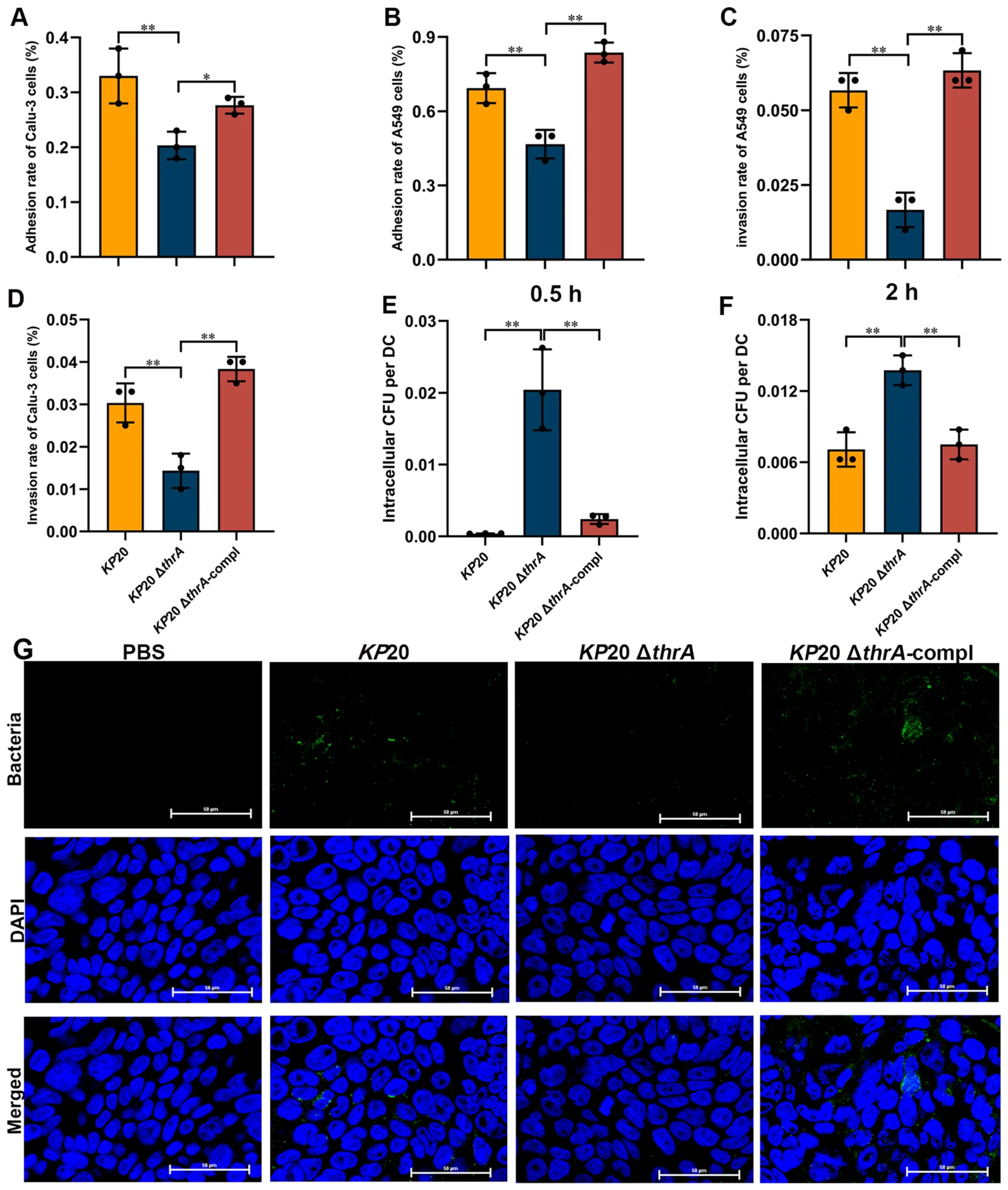
The adhesion and invasion rates of *K. pneumoniae* regarding Calu-3 and A549 cells and the rate of phagocytosis by DCs. The adhesion rates (**A**,**B**), invasion rates (**C**,**D**), and adhesion images (**G**) captured by CLSM regarding *KP*20, *KP*20 Δ*thrA*, and *KP*20 Δ*thrA*-compl, *K. pneumoniae* (Alexa Fluor 488; green) and nuclei (DAPI; blue) (bars: 50 µm). The phagocytosis rates of dendritic cells against *KP*20, *KP*20 Δ*thrA*, and *KP*20 Δ*thrA*-compl at 0.5 h (**E**) and 2 h (**F**). Data represent means ± SDs of three independent experiments. Pairwise comparisons were conducted between *KP*20 Δ*thrA* and *KP*20 and *KP*20 Δ*thrA* and *KP*20 Δ*thrA*-compl. ** *p* < 0.01, * *p* < 0.05.

Adhesion and invasion assays showed that *KP*20 Δ*thrA* had significantly fewer adherent and intracellular bacteria in Calu-3 and A549 cells than *KP*20 and *KP*20 Δ*thrA*-compl (Figure 5A–D).

Furthermore, we assessed the phagocytosis of three strains at an MOI of 100. At 0.5 and 2 h post-infection, relative to *KP*20 and *KP*20 Δ*thrA*-compl, more intracellular *KP*20 Δ*thrA* was recovered from DCs (Figure 5E,F).

### 3.7. Murine Infection Phenotype of the thrA Deletion Mutant

**Figure 6:**
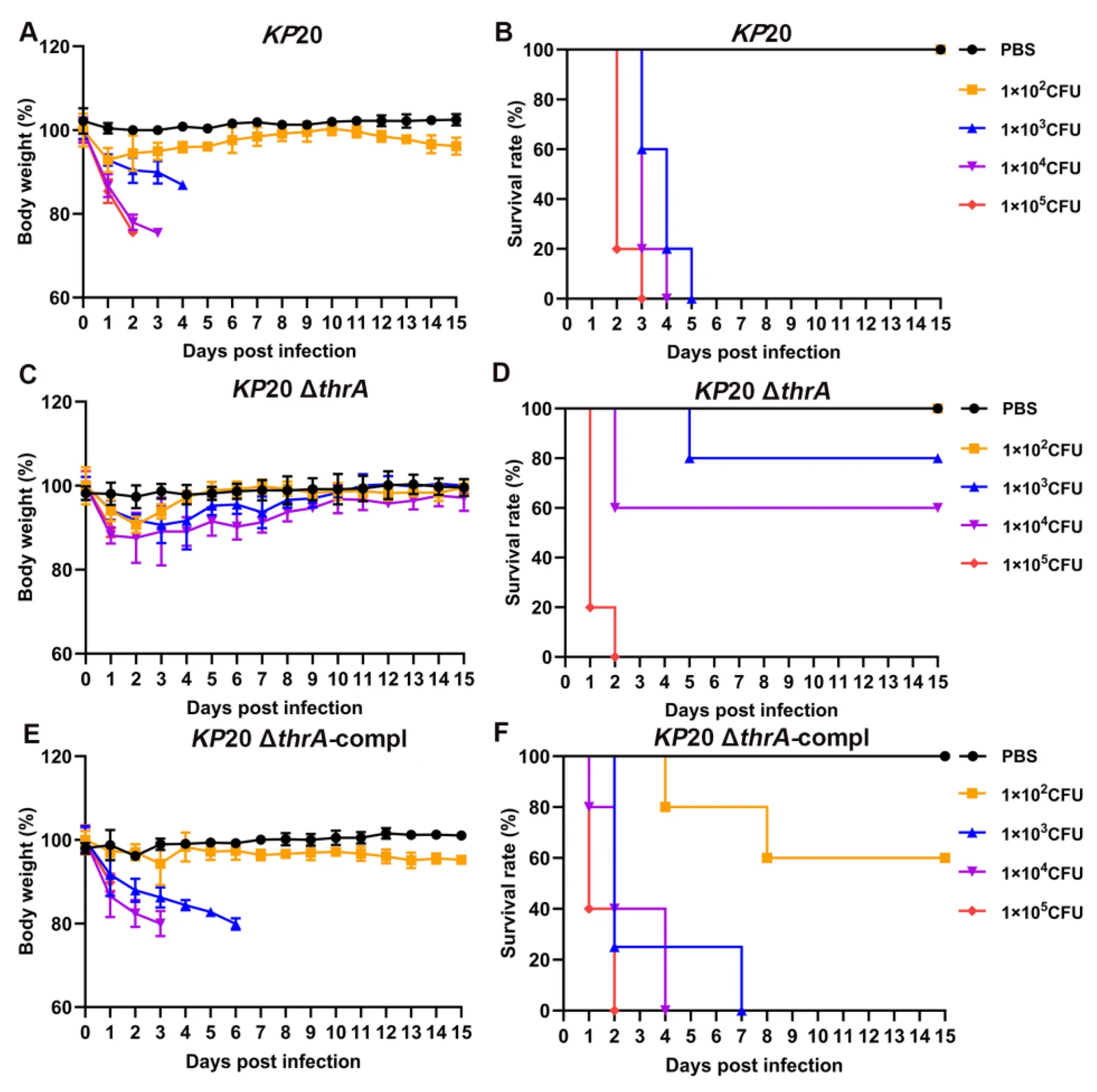
The bodyweight changes and survival rates among mice. Bodyweight changes in mice (*n* = 5) intratracheally infected with *KP*20 (**A**), *KP*20 Δ*thrA* (**C**), and *KP*20 Δ*thrA*-compl (**E**). The survival rates of mice (*n* = 5) intratracheally injected with *KP*20 (**B**), *KP*20 Δ*thrA* (**D**), and *KP*20 Δ*thrA*-compl (**F**). Data represent means ± SDs of five independent experiments.

To investigate the impact of the *thrA* gene on the pathogenicity of *K. pneumoniae* in vivo, mice were intratracheally challenged with three strains at various dosages. At high infectious doses (1 × 10^4^ and 1 × 10^5^ CFU), the mice in all experimental groups displayed obvious bodyweight loss during the observation period (Figure 6A,C,E). At a dose of 1 × 10^4^ CFU, the *KP*20 Δ*thrA* group maintained a 60% survival rate throughout the 15-day monitoring period, while the *KP*20 group exhibited 100% mortality by day 4 post-infection (Figure 6B,D). Similarly, at a low dose of 1 × 10^3^ CFU, the *KP*20 Δ*thrA-*infected mice retained an 80% survival rate (Figure 6D), whereas all mice in the *KP*20 group died within five days (Figure 6B). Consistent with the survival phenotypes, LD_50_ calculation using the modified Karber method demonstrated that the *KP*20 Δ*thrA* exhibited a 25-fold-higher LD_50_ value than the *KP*20 strain and a 63-fold-higher LD_50_ value than the complemented strain (Table 5).

**Figure 7:**
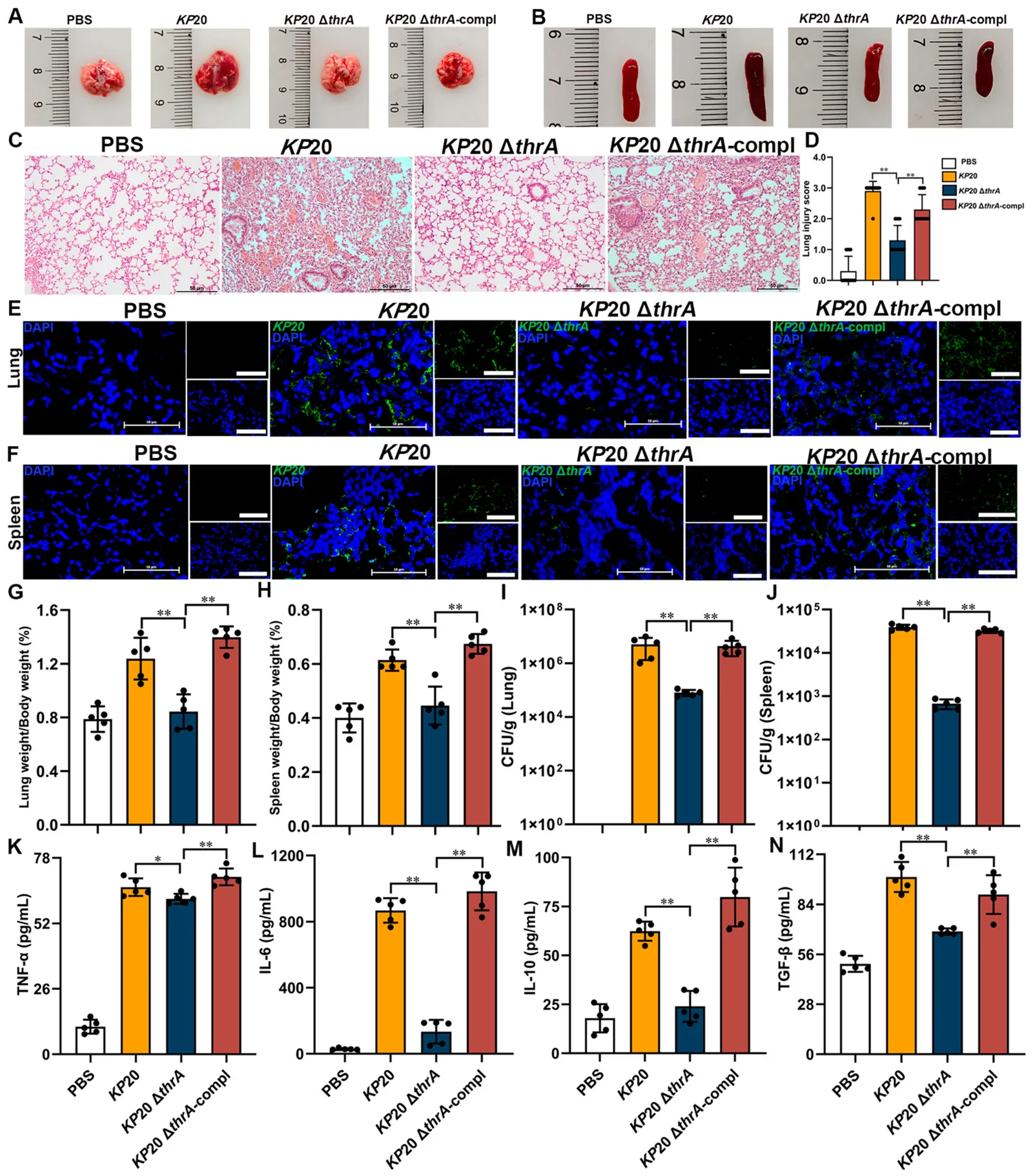
Evaluation of the pathogenicity of *K. pneumoniae*. (**A**,**B**) Pathology images of lungs and spleens (*n* = 5 per group). (**C**) Histopathological images of H&E-stained lung tissues (*n* = 5). (**D**) Histopathologic scores for the lungs. (**G**,**H**) Wet weights of the lungs and spleens of mice intratracheally injected with PBS, *KP*20, *KP*20 Δ*thrA*, and *KP*20 Δ*thrA*-compl at 24 h post-infection. The bacterial load in lungs (**E**,**I**) and spleens (**F**,**J**) and cytokine content (TNF-α (**K**), IL-6 (**L**), IL-10 (**M**), and TGF-β (**N**)) in the bronchoalveolar lavage fluid (BALF) in the lungs of mice intratracheally injected with PBS, *KP*20, *KP*20 Δ*thrA*, and *KP*20 Δ*thrA*-compl at 24 h post-infection. Data represent means ± SDs of five independent experiments. Pairwise comparisons were conducted between *KP*20 Δ*thrA* and *KP*20 and *KP*20 Δ*thrA* and *KP*20 Δ*thrA*-compl, ** *p* < 0.01, * *p* < 0.05. Bars: (**C**) 50 µm; (**E**,**F**) 50 µm.

To further verify the attenuated phenotype, pathological sections of mouse lung tissue were prepared 24 h after bacterial infection, and the degree of lung injury was evaluated via histological scoring. Pathological examination revealed that mice infected with *KP*20 and *KP*20 Δ*thrA*-compl exhibited significant pulmonary swelling and splenic necrosis (Figure 7A,B). H&E staining further showed severe pneumonia characterized by inflammatory cell infiltration and thickened alveolar walls (Figure 7C,D). In contrast, the *KP*20 Δ*thrA* group displayed significantly milder pulmonary lesions and lower pathological scores. To investigate the effect of *KP*20 Δ*thrA* on bacterial colonization in vivo, bacterial loads in mouse lung and spleen tissues were quantified. *KP*20 and *KP*20 Δ*thrA*-compl robustly colonized lung and spleen tissues, while the Δ*thrA* mutant displayed significantly reduced bacterial colonization in both organs (Figure 7E,F,I,J). Moreover, the *KP*20 Δ*thrA* group showed decreased wet weights of lungs and spleens (Figure 7G,H). These phenotypes were substantially restored in the complemented strain (Figure 7E–J).

Finally, we assessed the inflammatory response in mouse BALF. *KP*20 Δ*thrA* induced significantly lower levels of pro-inflammatory cytokines (TNF-α and IL-6) and anti-inflammatory factors (IL-10 and TGF-β) compared to the *KP*20 and *KP*20 Δ*thrA*-compl groups (Figure 7K–N). Collectively, these findings demonstrate that *thrA* substantially contributed to the virulence and colonization capacity of *K. pneumoniae* in a murine model.

### 3.8. qRT-PCR of Fim and Mrk Genes in the thrA Deletion Mutant

**Figure 8:**
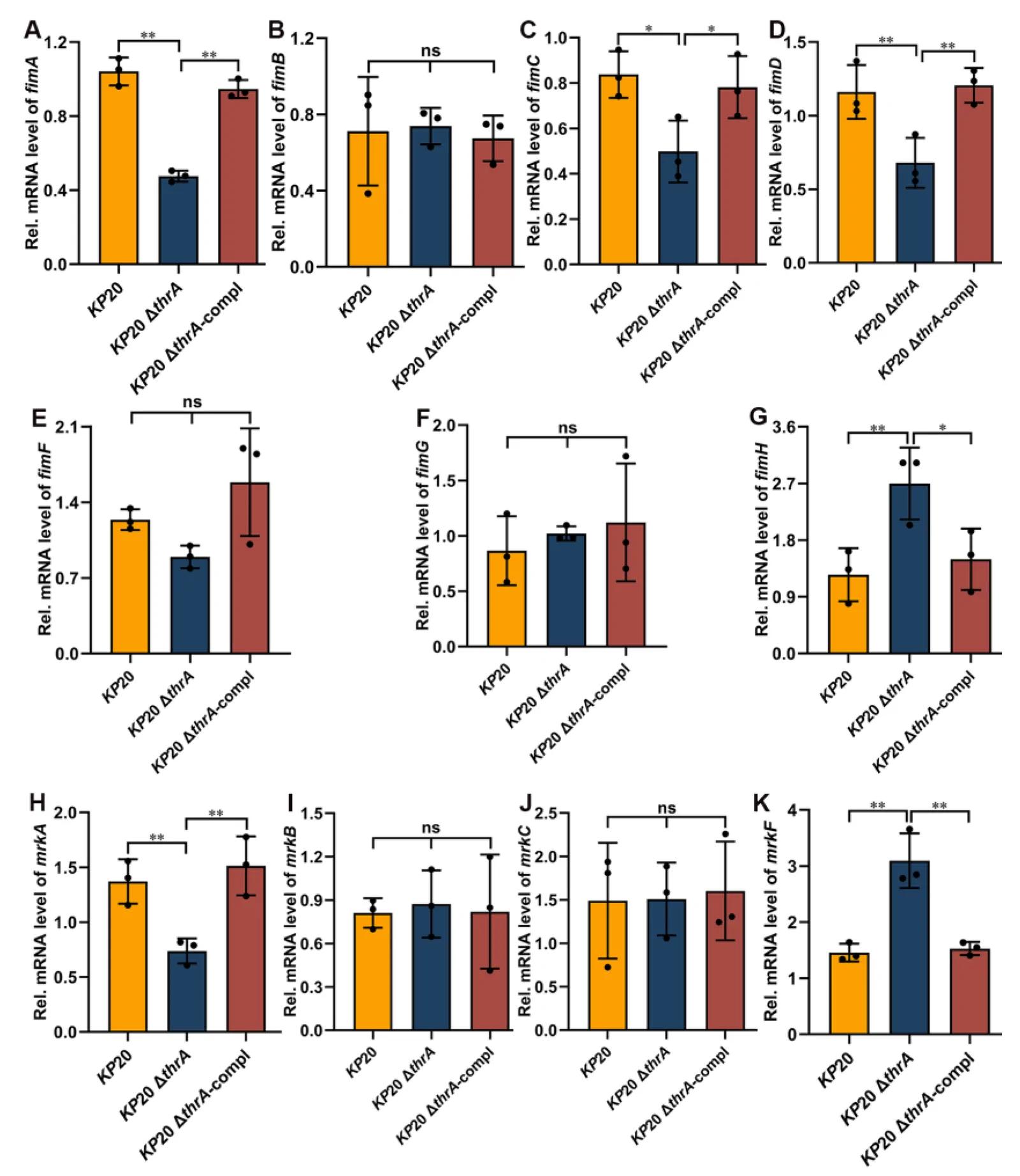
The transcriptional levels of biofilm-formation-related genes in *KP20*, *KP20* Δ*thrA*, and *KP20* Δ*thrA*-compl. The transcriptional levels of the *fimA* (**A**), *fimB* (**B**), *fimC* (**C**), *fimD* (**D**), *fimF* (**E**), *fimG* (**F**), *fimH* (**G**), *mrkA* (**H**), *mrkB* (**I**), *mrkC* (**J**), and *mrkF* (**K**) genes of *K. pneumoniae* were assessed through qRT-PCR. Data represent means ± SDs of three independent experiments. Pairwise comparisons were conducted between *KP*20 Δ*thrA* and *KP*20 and *KP*20 Δ*thrA* and *KP*20 Δ*thrA*-compl. ** *p* < 0.01, * *p* < 0.05, ns: *p* > 0.05.

To investigate the molecular basis of the biofilm formation defects, we performed qRT-PCR to analyze the transcriptional levels of the two major fimbria operons in *K. pneumoniae*. In the type I fimbria operon, the transcription levels of the major structural subunit gene (*fimA*) and the assembly related genes (*fimC* and *fimD*) were significantly downregulated in *KP*20 Δ*thrA* (Figure 8A,C,D). However, the transcription levels of regulatory and accessory genes (*fimB*, *fimF*, and *fimG*) remained unchanged (Figure 8B,E,F). The transcription level of the adhesin-encoding gene *fimH* was significantly upregulated (Figure 8G). Consistent with the differential expression pattern of type I fimbrial genes, the type III fimbrial operon also displayed distinct transcriptional changes. The major subunit gene *mrkA* was markedly downregulated in *KP*20 Δ*thrA* (Figure 8H), whereas the chaperone gene *mrkB* and usher gene *mrkC* showed no obvious transcriptional alterations (Figure 8I,J). Additionally, the assembly factor gene *mrkF* was significantly upregulated upon *thrA* deletion (Figure 8K). Overall, *thrA* deletion was associated with decreased transcription of *fimA*, *fimC*, *fimD*, and *mrkA* and increased transcription of *fimH* and *mrkF* under the tested conditions.

## 4. Discussion

*K. pneumoniae* is a Gram-negative, encapsulated opportunistic pathogen responsible for a wide spectrum of infections, including pneumonia, urinary tract infections, bacteremia, meningitis, and liver abscesses [29]. While traditionally associated with vulnerable populations such as neonates and the immunocompromised, the emergence of hypervirulent and multidrug-resistant strains has established *K. pneumoniae* as a major cause of community-acquired infections, posing a significant public health challenge [30]. A key determinant of its persistence, particularly in clinical settings, is its ability to form biofilms on medical devices like endotracheal tubes and catheters [31]. These biofilms facilitate chronic, recalcitrant infections that are increasingly difficult to eradicate [32]. To identify novel genes involved in *K. pneumoniae* biofilm formation, we constructed a mariner-based transposon mutant library in the clinical strain *KP*20. The screening of 600 mutant strains revealed four mutants whose biofilm formation was significantly reduced. The sequencing of these insertion sites identified two candidate genes associated with biofilm formation. The regulatory mechanisms of *thrA* in bacterial biofilm formation are poorly characterized, with limited relevant studies. Previous studies have verified that the *ornD* gene is involved in biofilm formation of *Yersinia pestis* [33]. Among them, *thrA* was selected for a detailed functional study via targeted gene knockout and genetic complementation assays.

The *thrA* gene encodes homoserine dehydrogenase (HSD), a component of the bifunctional aspartokinase/homoserine dehydrogenase complex that serves as a key rate-limiting enzyme in the aspartate metabolic pathway. This pathway is responsible for the biosynthesis of essential amino acids, including lysine, threonine, methionine, and isoleucine, in plants and microorganisms [34]. Beyond their fundamental nutritional value, these aspartate-derived amino acids are vital for stress adaptation. For example, lysine is commonly accumulated as a critical osmoprotectant and protective buffer to counteract various environmental stressors [35]. Consequently, disruptions in this pathway may impair cellular tolerance to external stressors. In *Candida albicans*, deletion of the HSD-encoding *hom6* gene reduced adhesion to polystyrene surfaces [36], whereas disruption of *thrC* significantly attenuated virulence in mice infected with *Staphylococcus aureus* [37]. Through the initial assays, we found that the loss of *thrA* does not affect growth rates in nutrient-rich LB medium, suggesting that *thrA* is not required for growth in nutrient-rich LB medium. This differs from the case for *Mycobacterium tuberculosis* [9], where *thrA* is related to growth rates, further highlighting the functional specificity of the *thr* gene family. String tests, mucoviscosity assays, capsule staining, and TEM revealed no morphological or structural differences between *KP*20 and *KP*20 Δ*thrA*, which showed comparable fimbrial density and morphology. Thus, *thrA* deletion barely alters capsule phenotypes or fimbrial biosynthesis in *K. pneumoniae*.

Collectively, these results indicate that *thrA* does not influence the physical properties of the bacterial cell surface or the production of ECM components independently of canonical capsular polysaccharide synthesis.

Given the clinical threat posed by multidrug-resistant *K. pneumoniae*, we further evaluated the antibiotic susceptibility of *KP*20 Δ*thrA* against a panel of 19 commonly used clinical antibiotics. The results revealed highly consistent resistance profiles among the three strains, indicating that *thrA* is not involved in regulating either intrinsic or acquired antibiotic resistance in *K. pneumoniae*. This functional characteristic differs from that of *Streptomyces clavuligerus*, in which the deletion of the HSD-encoding gene markedly enhances bacterial antibiotic resistance [38]. This interspecies discrepancy further underscores the distinct, species-specific functions of HSD family genes across different bacterial strains.

Interestingly, *KP*20 Δ*thrA* displayed prominent growth defects following 30 min exposure to both acidic and alkaline stress conditions. This pH-stress sensitivity may be associated with the impaired biofilm formation observed in *KP*20 Δ*thrA*. In contrast, the absence of obvious phenotypic changes under osmotic and oxidative stress indicates that *thrA* is not directly involved in these adaptive responses. It is also plausible that other functional genes could provide compensatory effects to offset the loss of *thrA* function under such stress conditions.

The growth restriction of *KP*20 Δ*thrA* under threonine deprivation further verifies the indispensable role of *thrA* in threonine anabolism. Stepwise growth recovery with increasing threonine concentrations demonstrated that exogenous threonine could compensate for the loss of endogenous synthesis. The 200 μM minimal rescue concentration defines the minimum extracellular threonine supply necessary to meet the metabolic requirements for *KP*20-level proliferation of this mutant.

Further confirmation was obtained through 96-well plate biofilm phenotyping assays, and SEM revealed that *KP*20 Δ*thrA* exhibited significantly reduced biofilm formation capacity compared to *KP*20 and *KP*20 Δ*thrA*-compl. Notably, the complemented strain restored biofilm formation to levels that exceeded those of the wild-type strain. This “gain-of-function” phenotype is likely attributable to a gene dosage effect. The complementation vector, pACYC184, utilizes the p15A origin of replication, which typically maintains a low-to-medium copy number of approximately 10–15 copies per cell in *K. pneumoniae*. Meanwhile, Supplementary Materials S4 and Figure S3 confirmed that pACYC184 drives robust target gene overexpression, strengthening the validity of our complementation results. Compared to the single chromosomal copy of *thrA* in the WT, this moderate overexpression likely enhances the availability of HSD, thereby increasing the metabolic flux toward biofilm formation. Liu et al. [39] found that upregulation of the biofilm-promoting type I fimbrial gene *fimA* in *E. coli* simultaneously triggers the threonine biosynthetic operon *thrABC*, indicating biofilm formation requires carbon flux for threonine synthesis, and impaired threonine metabolism inhibits fimbriae and matrix production. This supports our finding that *thrA* deletion blocks endogenous threonine synthesis in *KP*20, causing insufficient precursors to assemble adhesive structures and drastically reducing biofilm biomass.

Adhesion and invasion assays performed in Calu-3 and A549 epithelial cells demonstrated that the Δ*thrA* mutant exhibited significantly impaired interactions with host epithelial cells. This phenotypic alteration is consistent with a previous report showing that the hom6 gene of *Candida albicans* governs host adhesion through the threonine metabolic pathway [36]. Furthermore, *KP*20 Δ*thrA* exhibited markedly higher susceptibility to phagocytosis by DCs, indicating that *thrA* contributes to the anti-phagocytic capacity of *K. pneumoniae*. The enhanced susceptibility of *KP*20 Δ*thrA* to DC-mediated phagocytosis renders the bacterium more vulnerable to innate immune clearance. Similar regulatory patterns have been observed for other amino acid metabolism-associated enzymes; for instance, the membrane-modifying enzyme *LysX* confers mycobacterial resistance to cationic antimicrobial peptides [40]. These findings collectively highlight that amino-acid-linked metabolic pathways may influence host–pathogen interactions.

The in vivo significance of *thrA* was assessed in a murine pneumonia model. The LD_50_ of *KP*20 Δ*thrA* was 25-fold higher than that of *KP*20 and 63-fold higher than that of the *KP*20 Δ*thrA*-compl, indicating that *KP*20 Δ*thrA* significantly attenuates bacterial virulence. *KP*20 Δ*thrA-*compl displayed only a non-significant numerical trend of increased virulence versus *KP*20, evidenced by its modestly reduced LD_50_. This subtle trend could stem from pACYC184-mediated gene overexpression within the complemented strain. The mice infected with the mutant showed significantly higher survival rates and reduced weight loss. These results demonstrate that *thrA* plays an important role in the pathogenicity of *K. pneumoniae*, likely by supporting bacterial adaptation and fitness within the host. These findings are consistent with previous studies, wherein the deletion of the *thrC* of homocysteine dehydrogenase increased survival in *Xanthomonas*-infected rice [41]. Lung and spleen wet weight are a significant indicator of the degree of lung and spleen inflammation, and pathological changes directly reflect bacteria’s ability to damage these tissues. Our study revealed that *KP*20 Δ*thrA* resulted in significantly milder lung and spleen tissue damage and lower wet weights than *KP*20 and *KP*20 Δ*thrA*-compl. These findings indicate that *thrA* contributes to the ability of *K. pneumoniae* to damage these tissues. Furthermore, bacterial loads in the lungs and spleens were significantly lower in the *KP*20 Δ*thrA*-infected mice, suggesting that *thrA* deficiency impairs colonization and systemic dissemination. The reduced inflammatory cell infiltration and lower levels of both pro- and anti-inflammatory cytokines in the BALF suggest that the mutant failed to establish a robust infection, thereby triggering a more muted immune response. Consistent with observations made regarding *Mycobacterium tuberculosis*, where disruption of the aspartate synthesis pathway (via *thrA* mutation) substantially attenuates virulence [41], our findings demonstrate that *thrA* is vital for *K. pneumoniae*’s pathogenicity. Collectively, these data suggest that *thrA* may contribute to maintaining the bacterial fitness required to trigger and sustain host inflammatory responses.

To examine transcriptional changes associated with impaired biofilm formation and adhesion, we quantified the expression of the type I (fim) and type III (mrk) fimbrial operons by qRT-PCR. Although transcripts encoding the type I fimbrial main structural subunit *fimA*, periplasmic chaperone *fimC*, and outer-membrane usher *fimD* were significantly downregulated, the terminal adhesin-encoding gene *fimH* exhibited prominent upregulation, whereas the regulatory gene *fimB* retained steady expression without obvious variation. For the type III fimbrial (*mrk*) cluster, expression of the major structural gene *mrkA* was repressed, while that of the accessory tip subunit gene *mrkF* was markedly elevated; by contrast, transcriptional levels of the regulatory gene *mrkB* and chaperone gene *mrkC* remained unaltered.

These transcriptional changes coincided with reduced epithelial adhesion and biofilm formation; however, qRT-PCR alone cannot establish causal mediation or functional compensation. Accumulating evidence from prior investigations is identifying type III fimbriae as critical virulence factors mediating the adhesion and biofilm development of *K. pneumoniae* [42]. As the predominant structural subunit of type III fimbrial shafts, *MrkA* substantially promotes bacterial biofilm maturation, especially on abiotic substrates [43]. Compared with *KP*20, *KP*20 Δ*thrA* displays marked defects in epithelial adhesion and biofilm formation, which may be related to the downregulation of *mrkA*. Although *thrA*-associated pathways may warrant exploration as antimicrobial or vaccine-related targets, the present data do not establish *thrA* as a validated vaccine antigen or therapeutic target.

This study has several limitations. Differences in global regulatory factors (CRP, Rcs, etc.) may indirectly affect the transcription of the *fim* and *mrk* genes; however, this study has not verified the regulatory relationship between *thrA* and these factors. Although deletion of *thrA* is associated with altered transcription of *fim* and *mrk*, the underlying regulatory mechanism and its effects on fimbrial protein abundance still remain unclarified. While our results clearly demonstrate the restoration of the *KP*20 phenotype via *KP*20 Δ*thrA*-compl, we acknowledge that an empty vector control (Δ*thrA* + pACYC184) was not included in all the assays. Therefore, we cannot entirely rule out minor contributions of plasmid maintenance or antibiotic selection to the observed metabolic state. However, given that the complementation resulted in a significant ‘gain-of-function’ (particularly in biofilm OD_550_ levels and LD_50_) rather than a further reduction in fitness, it is highly probable that the observed effects are *thrA*-specific.

Another limitation of this study is the absence of a standard reference strain for comparison. We used clinical isolate *KP*20 owing to its strong biofilm-forming ability and clinical relevance, yet the lack of a universal reference strain hinders direct cross-study comparisons with strains of distinct genetic backgrounds. Nevertheless, as this work primarily aimed to characterize thrA function, we prioritized isogenic comparisons among *KP*20, *KP*20 Δ*thrA*, and *KP*20 Δ*thrA*-compl. This strategy ensures observed phenotypic alterations stem solely from the *thrA* locus, eliminating confounding effects arising from the widespread genomic plasticity of *K. pneumoniae*. In addition, the animal experiment yielded broad 95% CIs (e.g., 2220–28,500 CFU for *KP*20 Δ*thrA*), spanning over one order of magnitude. Given the limited statistical precision, future studies will use larger animal groups to improve statistical resolution.

Finally, discrepancies exist between qRT-PCR data and TEM observations of fimbriae in this study. TEM only provides qualitative morphological evaluation and cannot discriminate type I and type III fimbriae, since *K. pneumoniae* carries diverse fimbrial gene clusters [44]. Wilksch et al. [45] reported discrepancies between *mrkA* transcription, MrkA protein levels and functional type III fimbrial biogenesis. The maturation and surface presentation of type III fimbriae require multiple accessory factors: MrkB mediates periplasmic folding, whereas MrkC drives polymerization and secretion. Thus, qRT-PCR merely detects transcriptional fluctuations of fimbrial genes and cannot directly reflect the abundance of assembled surface fimbriae. Further quantitative protein detection and structural characterization are required.

## 5. Conclusions

In conclusion, this study identifies *thrA* as an important factor for *K. pneumoniae* biofilm formation and in vivo virulence. The molecular mechanisms by which HSD, and *thrA* in particular, coordinate such diverse physiological processes remain a subject of active investigation. In the development of pertussis vaccines, HSD has been identified as a novel therapeutic target and immunogenic candidate for enhancing therapeutic and preventive measures against *Bordetella pertussis* [46]. While the exact signaling intermediates between aspartate metabolism and fimbrial expression remain to be fully elucidated, the significant attenuation of the *KP*20 Δ*thrA* mutant highlights its potential as a target for therapeutic intervention. Further studies will elucidate the regulatory mechanisms of *thrA* in modulating the metabolic pathways of *K. pneumoniae*.

## Figures and Tables

**Table 2:** Primers designed and used in this study.

Primers	Primer Sequence (5′ to 3′)
ARB1	GGCCACGCGTCGACTAGTACNNNNNNNNNNACGCC
P7-1	TATAATGTGTGGAATTGTGAGCGG
ARB2	GGCCACGCGTCGACTAGTAC
P7-2	ACAGGAAACAGGACTCTAGAGG
XbaI-K*_thrA_*-A	GCCTCTAGAAGCGTCCAAGATTCTGCAG
K*_thrA_*-B	TAAGCATGTTCTGCACGGCAAGGTAAAGTCTTGCGCTACGTC
K*_thrA_*-C	GACGTAGCGCAAGACTTTACCTTGCCGTGCAGAACATGCTTA
XhoI-K*_thrA_*-D	GCTCTCGAGGGTAAGAACCATGCGAGTGT
HindIII-K*_thrA_*-F	GCTTCTAGAGTATGCTGTTCTATGGGCTGGT
BamHI-K*_thrA_*-R	GCTAAGCTTCCGACGCTCATATTGGCACT
*thrA*-F	TCTTCGCTGGATTACTAT
*thrA*-R	CGTTAGTGTCATAGAGGA
*mrkA*-F	CATGACTGCTGCCCATGCAG
*mrkA*-R	CTTCAGTCAGGGTTACCGGAG
*mrkB*-RT-F	AGGCCTGGCTGGATAACGG
*mrkB*-RT-R	AATGTAGAACGGCGTCGGGT
*mrkC*-RT-F	GCCCGGG AAGATTAAGCACC
*mrkC*-RT-R	CTTCAGCAGCCAGCCGTCC
*mrkF*-RT-F	ATGAAGGGATTGCCGAAAAA
*mrkF*-RT-R	GCTCCATCCGGCAAGGTA
*fimA*-RT-F	GTGGATGCCGGCTCTATCGATC
*fimA*-RT-R	CGCTTTGGTGGCTACCGTAG
*fimB*-RT-F	GACAGTAACCCAATCCCTTTCG
*fimB*-RT-R	TTTTTCCATCAGCCACGCC
*fimC*-RT-F	AGGATGTGTGCTTTTCGCC
*fimC-*RT-R	GCATTTTCCACCCATGACTG
*fimD*-RT-F	GCGAACATCAGGGGCAGTC
*fimD*-RT-R	CGGTAAGTGGTATCGGCAAAG
*fimF*-RT-F	TTTAGCCTGATCGGTGCGG
*fimF*-RT-R	CGTTCCCTGGTTTTTGTAATAGC
*fimG*-RT-F	CTATGGCGGTGTGCTGTCG
*fimG*-RT-R	GGGTTTATCGGTCCGTGAATC
*fimH*-RT-F	TGACGGCGTTTGAACAGGA
*fimH*-RT-R	GTGCGGCAGAAAGGTCTGG
*KP*-*16S*-RT-F	ATGACCAGCCACACTGGAAC
*KP*-*16S*-RT-R	CTTCCTCCCCGCTGAAAGTG

**Table 3:** Identification and characterization of insertion sites in biofilm-formation mutants of *KP*20.

Mutant	Gene Name	Description
M70	*thrA*	Homoserine dehydrogenase
M81	*ornD*	Ornithine decarboxylase
M117	*ornD*	Ornithine decarboxylase

**Table 4:** Antimicrobial susceptibility profiles of 3 strains of *K. pneumoniae*.

Antibiotic	Strain		
	*KP*20	*KP*20 Δ*thrA*	*KP*20 Δ*thrA*-Compl
CIP	I	I	I
NOR	S	S	S
OFX	S	S	S
C	S	S	R
TE	R	R	R
DX	R	R	R
MI	R	R	R
K	S	S	S
GM	S	S	S
SM	I	I	I
AK	S	S	S
IPM	I	I	I
MEM	S	S	S
AT	S	S	S
FOX	S	S	S
CTR	S	I	S
CFM	S	S	S
CZ	S	S	S
AM	R	R	R

R—resistant; S—susceptible; I—intermediate; C—chloramphenicol; AK—amikacin; CIP—ciprofloxacin; DX—doxycycline; GM—gentamicin; AM—ampicillin; K—kanamycin; NOR—norfloxacin; FOX—cefoxitin; MEM—meropenem; IPM—imipenem; AT—aztreonam; OFX—ofloxacin; TE—tetracycline; MI—minocycline hydrochloride; SM—streptomycin; CTR—ceftriaxone sodium; CFM—cefixime; CZ—cefazolin.

**Table 5:** The LD_50_ results obtained from the mice.

Strain	Challenge Dose (CFU)				LD_50_ (CFU)	95% Confidence Interval (CFU)
	1.0 × 10^2^	1.0 × 10^3^	1.0 × 10^4^	1.0 × 10^5^		
*KP*20	0/5	5/5	5/5	5/5	3.16 × 10^2^	1.31 × 10^2^–7.62 × 10^2^
Δ*thrA*	0/5	1/5	2/5	5/5	7.94 × 10^3^	2.22 × 10^3^–2.85 × 10^4^
Δ*thrA*-compl	2/5	5/5	5/5	5/5	1.26 × 10^2^	0.47 × 10^2^–3.38 × 10^2^

## Data Availability

The original contributions presented in this study are included in the article/Supplementary Material. Further inquiries can be directed to the corresponding authors.

## Supplementary Materials

The following supporting information can be downloaded at: https://www.mdpi.com/article/10.3390/microorganisms14081751/s1, Figure S1. After 24 h static incubation of *K. pneumoniae* in Nutrient Broth at 37 °C, absorbance was measured at 600 nm; Figure S2. Agarose gel electrophoresis graph of the arbitrary PCR. M: DL2000 DNA marker; 1, 2, 3, 4, 5: M7, M70, M70, M81, and M117 result of PCR round 1; 6, 7, 8, 9, 10: M7, M70, M70, M81, and M117 result of PCR round 2; Figure S3. The transcriptional levels of *thrA* gene in *KP*20, *KP*20 Δ*thrA*, and *KP*20 Δ*thrA*-compl.

## Abbreviations

The following abbreviations are used in this manuscript:

HSD	homoserine dehydrogenase
*KP*	*K. pneumoniae*
