# Supplementary material for: The Homoserine Dehydrogenase thrA Significantly Impairs Biofilm Formation, Stress Adaptation, and Virulence in Klebsiella pneumoniae
Source: Microorganisms. 2026 Aug 9;14(8):1751. doi: 10.3390/microorganisms14081751 (PMC13515714; doi:10.3390/microorganisms14081751)
Supplement: Supplementary file 1 [file microorganisms-14-01751-s001.zip › microorganisms-4414888-supplementary.pdf]

Supplementary Methods S1

Construction and screening of the mariner transposon mutant: A transposon mutant library was generated using the mariner-based transposon system. Briefly, the donor strain (*E. coli* S17-λpir carrying the transposon delivery plasmid pBT20) and the recipient strain (*KP*20-pKD46-Kan) were grown in LB broth at 37°C with shaking at 220 r/min. Subsequently, the bacterial cultures were adjusted to an OD_{600} of 0.4 for the donor and 0.2 for the recipient. The two suspensions were mixed at an equal volumetric ratio, and 50 μL aliquots were spotted onto LB agar plates. After incubation at 37°C for 12 h to facilitate bacterial conjugation, the mating bacterial mixtures were harvested from the agar surface and resuspended in fresh LB broth. Serial dilutions of the resuspended mixtures were then spread onto LB agar plates containing gentamicin (10 μg/mL) and kanamycin (50 μg/mL) for transconjugant selection.

Supplementary Methods S2

Identification of transposon insertion sites: Transposon mutants were screened for reduced biofilm formation ability via a 96-well microplate crystal violet staining assay. Briefly, individual mutants were cultured overnight in LB broth supplemented with 50 μg/mL gentamicin at 37°C with shaking at 220 r/min. The overnight cultures were adjusted to an OD_{600} of 0.8, then this culture fluid was diluted 1: 10 with 1/10 Nutrient Broth diluent and then transferred to a 96-well U-shaped polystyrene plate (200 μL/well) for static cultivation at 37°C for 24 h. Following incubation, planktonic cells were removed, and the plates were gently washed with ddH_2O to eliminate non-adherent bacteria. The formed biofilms were then stained with 200 μL of 0.4% (w/v) crystal violet (CV) solution for 30 min at room temperature. Following staining, excess unbound CV was thoroughly washed away with ddH_2O, and the plates were air-dried naturally. The CV bound to biofilm biomass was solubilized using 200 μL of absolute ethanol per well, and the absorbance value at 550 nm (OD_{550}) was measured with a microplate spectrophotometer. The WT strain was set as the blank control in all parallel assays. Mutant strains exhibiting a statistically significant reduction in biofilm formation (lower OD_{550} values) compared with the WT strain were selected for subsequent molecular and phenotypic characterization.

Supplementary Methods S3

Construction and verification of *KP*20 Δ*thrA* and *KP*20 Δ*thrA*-compl: The transposon insertion sites were identified using arbitrary primed PCR. The identification procedure involved two sequential PCR reactions. For the first-round PCR, genomic DNA extracted from biofilm-deficient mutant strains was used as the template, with a transposon-specific primer (P7-1) and an arbitrary primer (ARB1) applied for amplification (Table 2). The thermal cycling conditions were set as follows: initial denaturation at 95°C for 5 min; 6 cycles of 95°C for 30 s, 30°C for 30 s, and 72°C for 1.5 min; 30 cycles of 95°C for 30 s, 45°C for 30 s, and 72°C for 2 min; and a final extension at 72°C for 4 min. The purified products obtained from the first-round PCR were used as templates for the second-round PCR, which was performed using a nested transposon-specific primer (P7-2) and ARB2 (Table 2). The amplification program for the second PCR consisted of an initial denaturation at 95°C for 1 min, 30 cycles of 95°C for 30 s, 52°C for 30 s, and 72°C for 2 min, and a final extension at 72°C for 4 min. The final purified PCR products was sequenced with the P7-2 primer. All primer synthesis and DNA sequencing were performed by Sangon Co. Ltd. (Shanghai, China).

Supplementary Methods S4

qRT-PCR was performed following the protocols described in Section 2.15. Briefly, bacterial RNA was extracted using the TRIzol method, followed by cDNA synthesis and SYBR Green-based amplification. The *16S* rRNA gene was used for internal normalization, and the $2^{-\Delta\Delta Ct}$ method was adopted to calculate relative gene transcription level. This assay was used to quantify *thrA* transcription for validating the complemented strain. Primer sequences are listed in Table 2.

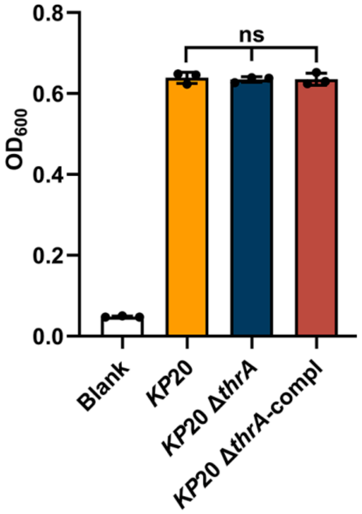

Figure S1. After 24 h static incubation of *K. pneumoniae* in Nutrient Broth at 37°C, absorbance was measured at 600 nm.

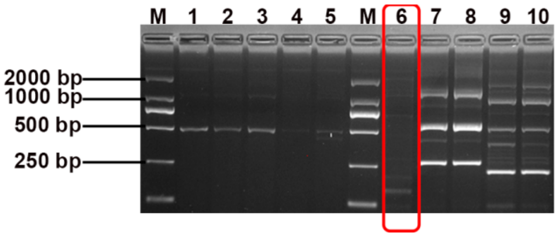

Figure S2. Agarose gel electrophoresis graph of the arbitrary PCR.

M: DL2000 DNA marker; 1, 2, 3, 4, 5: M7, M70, M70, M81, and M117 result of PCR round 1;

6, 7, 8, 9, 10: M7, M70, M70, M81, and M117 result of PCR round 2.

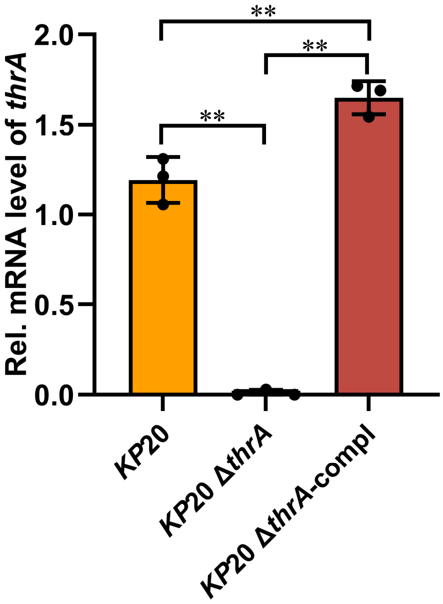

Figure S3. The transcriptional levels of *thrA* gene in *KP*20, *KP*20 Δ*thrA*, and *KP*20 Δ*thrA*-compl.
